# Potential Solutions Using Bacteriophages against Antimicrobial Resistant Bacteria

**DOI:** 10.3390/antibiotics10121496

**Published:** 2021-12-06

**Authors:** Aryan Rahimi-Midani, Seon-Woo Lee, Tae-Jin Choi

**Affiliations:** 1 Department of Applied Bioscience, Dong-A University, Busan 49315, Korea; Aryan1372@dau.ac.kr (A.R.-M.); seonlee@dau.ac.kr (S.-W.L.); 2 Department of Microbiology, Pukyong National University, Busan 48513, Korea

**Keywords:** bacteriophages, phage therapy, phage biocontrol, combine modality therapy

## Abstract

Bacteriophages are viruses that specifically infect a bacterial host. They play a great role in the modern biotechnology and antibiotic-resistant microbe era. Since the discovery of phages, their application as a control agent has faced challenges that made antibiotics a better fit for combating pathogenic bacteria. Recently, with the novel sequencing technologies providing new insight into the nature of bacteriophages, their application has a second chance to be used. However, novel challenges need to be addressed to provide proper strategies for their practical application. This review focuses on addressing these challenges by initially introducing the nature of bacteriophages and describing the phage-host-dependent strategies for phage application. We also describe the effect of the long-term application of phages in natural environments and other bacterial communities. Overall, this review gathered crucial information for the future application of phages. We predict the use of phages will not be the only control strategy against pathogenic bacteria. Therefore, more studies must be done for low-risk control methods against antimicrobial-resistant bacteria.

## 1. Introduction

Bacteria are among the simplest forms of life, numerous and inhabitants of almost any environment, including places with acidic and hot conditions [1]. The prokaryotic-eukaryotic concept and molecular technologies distinguish bacteria from each other and separate them as an independent evolutionary domain from other forms of life [2]. The cell wall structure of bacteria and Gram staining method divide bacteria into two groups. Group one includes Gram-positive bacteria that have a thick layer as a shell whereas, in group two, Gram-negative bacteria have a relatively thinner layer with an extra layer over and above with pores [3]. Bacterial cell growth depends on the availability of proper temperatures, pH conditions and nutrients. Different from beneficial bacteria, pathogenic ones have the property to cause diseases. Due to the economic importance of pathogenic bacteria, more studies have focused on this type [4].

Infectious diseases cause huge crop losses and severe animal and human diseases worldwide every year. For instance, phytobacteria, by causing diseases on a wide range of plants, directly impact the food supply. In addition, other pathogenic bacteria also cause of a high mortality rate in humans and animals [5,6,7]. Recently, studies showed that many pathogenic bacteria share a common mechanism to survive and live in their host cells. Bacterial cells attach to host cells and use the host’s systems to grow and reproduce [8,9]. Hence, insights into mechanisms of bacterial pathogenicity can lead us to understand how available control agents can stop bacteria from attachment and growth on host cells, and also provide information for better targeting the pathogen upon infection.

Bacterial pathogenicity occurs through virulence factors, route of entry, and surpassing the host defense mechanisms. Initially, adherence of bacteria to the surface of the host cell allows the pathogen to further interact with the host [10]. Mucus flow and ciliary movements in animal cells, cell walls, epidermis, and cuticle in plant cells are among the first mechanical barriers against microbial pathogens. Bacterial cells after passing these mechanical barriers can adhere to their host cells [11]. The cell surface of the bacterial cell has a direct impact on its adhesion to other surfaces. These pathogens commonly express “adhesins” which are made up of polysaccharides or polypeptides. The outer membrane of bacteria mediates the adhesion to the host cells. For instance, uropathogenic *Escherichia coli* (UPEC) colonizing the urinary tract and causing infections in the kidneys, has P pili on its surface [12]. In addition, the bacterial cell wall composition containing proteins and polysaccharides help bacteria during their attachment. For instance, enteropathogenic *Yersinia pseudotuberculosis* has a protein called YadA anchored on the surface of the outer membrane. YadA forms a capsule-like cover on the surface of bacteria protecting the bacteria from the defense mechanisms of host cells [13].

To date, many strategies for controlling bacterial diseases have been introduced and used. However, since the 1920s until the present, antibiotics have been mainly used to control bacterial diseases [14,15]. Antibiotics as a widely used chemical control have been developed since 1920 while other chemicals with control activity have been used periodically and or on a smaller scale. Since then, understanding the mechanism of action and discovery of antibiotics led to the large production of antibiotics [16]. However, the overuse of antibiotics in the past decades has resulted in the incidence of antibiotic-resistant bacteria. Bacterial cells adopt resistant genes and evolve and survive in the presence of antibiotics. Antibiotic resistance started to increase immediately after the introduction of antibiotics. Mechanisms to resist antibiotics are common among most bacterial strains [17]. The mainly mechanisms involve limiting the uptake of a drug, modifying the target site and inactivating a drug. For instance, methicillin-resistant *Streptococcus aureus* (MRSA) is known to be resistant to multiple antibiotics and causes serious infections in humans. The PBP2a protein produced by MRSA binds to β-lactam antibiotics and inhibits the antibiotics’ interaction with bacterial cell walls [18]. On the other hand, *Aeromonas* species carrying the gene strAB can encode enzymes that inactivate the streptomycin molecule through phosphorylation or adenylation [19].

Antibiotic-resistant bacteria cause a wide range of diseases worldwide. Antibiotic-resistant bacteria are also reported in plants, human and animal pathogens. However, due to the mass production of antibiotics, the first solution against bacterial diseases is still the application of antibiotics [20] and therefore novel methods for controlling bacterial infections are highly necessary.

## 2. Bacteriophages

**Figure 1:**
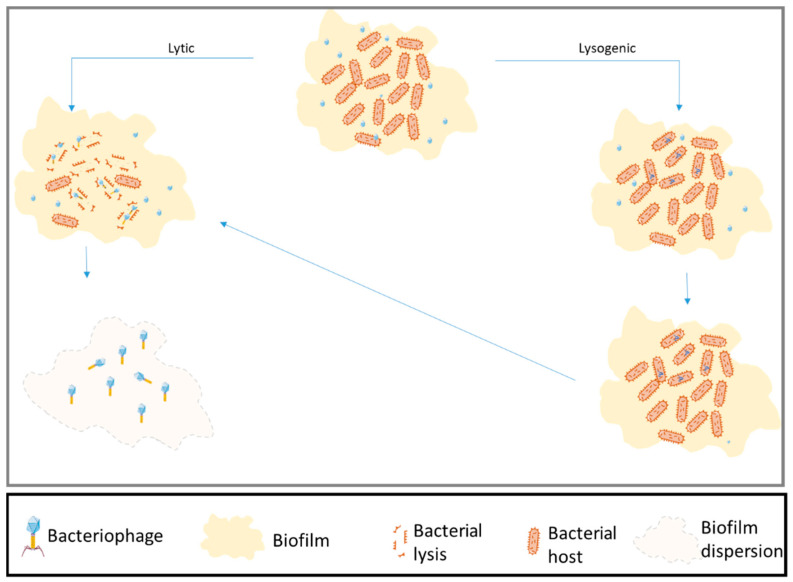
Schematic view of bacteriophage life cycle. Bacteriophages infecting bacterial strains in a biofilm. Unlike other control methods, phages are able to penetrate the biofilm to cause death in the host.

Bacteriophages, discovered in 1915, are the most numerous entities on Earth and are found in extreme environments as well as in the ocean, soil, and the human body [21,22]. They replicate using the bacterial host’s replication system, destroy the host cell (lytic cycle), or reside in the bacterial genome (lysogenic state). To initiate their biological infection cycle, phages attach to specific bacterial receptors; therefore, each bacteriophage is specific to one bacterial taxon. After attachment, the bacteriophage injects its genome into the cell and requisitions the bacterial replication machinery to produce phage genomes and proteins. These assemble inside the host cell. Because phages are much smaller than their host cell, one bacterial cell can produce hundreds to thousands of new phages [23]. Newly produced bacteriophages lyse the host cell and are released into the extracellular environment (Figure 1).

Pathogenic bacteria aggregate and become embedded in a matrix of extracellular polymeric substances (EPS) known as a biofilm. The formation of biofilms promotes survival of bacteria in the presence of antibiotics and biocides [24]. This phenomenon is mediated by slow penetration of antibiotics, adaptive stress response, or promotion of mutations that confer antibiotic resistance [25]. However, bacteriophages can interact with pathogenic bacteria even in biofilms. They penetrate the biofilm, replicate within bacterial cells, and produce enzymes that destroy extracellular polymeric substances [26]. For instance, the bacteriophage CT-PA reduces the number of multidrug-resistant *Pseudomonas aeruginosa* in biofilms at a concentration of 10^8^–10^10^ PFU/mL within 24–48 h [27]. This is because of the small size of the bacteriophage and a broad host range that enable the phage to penetrate the biofilm of *P. aeruginosa* and display a suitable anti-biofilm action [28].

The use of bacteriophages to control bacterial diseases essentially ceased with the advent of antibiotics. Thereafter, few studies have evaluated the ability of bacteriophages to control bacterial infection; bacteriophages were typically used only for molecular and genetic studies [29]. Sequencing of bacteriophage genomes has led to the realization of their ecological importance. The emergence of multiple-resistant bacteria has made bacteriophages more suitable for controlling bacterial infections. However, use of bacteriophages has advantages and disadvantages, as described in this part of our review where we study the available information on phages as therapeutic agents.

### 2.1. Advantages of Bacteriophages

#### 2.1.1. Self-Replication and Auto Dosing

Unlike antibiotics, bacteriophages replicate at the site of infection as long as susceptible bacteria are present. Bacteriophage self-replication not only promotes resolution of infections but also prevents further infection by pathogenic bacteria [30]. In addition, unless exposed to mechanical damage (like UV), phages are able to remain infective and viable outside of the host cell. This property of phages with auto dosing can be used to eradicate pathogens during food processing. On the other hand, auto-dosing can interfere with the co-application of other pharmaceutical drugs [31]. In fact, to avoid this issue, application of bacteriophage in low doses improves the safety of application. In addition, available information on the nature of bacteriophages like its one-step growth provides an important insight into the final number of produced phages after one cycle of infection [32,33]. Therefore, this property is a great benefit to avoid any side effects and a better strategy for the combined application of phages with other drugs such as antibiotics.

#### 2.1.2. Narrow and Specific Host Range

Individual bacteriophages are capable of infecting single or multiple strains of a bacterial species [34]. This property both promotes control of pathogens and sparing of the normal flora. Because antibiotics kill both pathogenic and nonpathogenic bacteria, phages are more suitable for controlling bacterial infections. For example, use of antibiotics against *Clostridium difficile* infection causes collateral damages while use of bacteriophages cocktail control the colonization of bacteria and the disease symptoms [35].

Narrow and specific host ranges may also be challenging for development of phage therapy. For instance, compared to broad activity of antibiotics against multiple bacteria, the application of phages will remove only the host bacterium without infecting the resistant strains. Therefore, in order to control a pathogenic bacterium, multiple numbers of phages must be used. To overcome this challenge, the preparation of a bacteriophage bank against each pathogenic bacterium is a must. Unlike antibiotics, phages do not affect the normal microbial flora and the use of phage cocktails will control the pathogenic bacteria as main advantages over the antibiotics [36].

#### 2.1.3. Ease of Isolation

Phages are readily isolated because of their ubiquity and abundance [37]. Because bacteriophages evolve with their host, new broad-host-range phages can be generated using cocktails of host species and environmental samples. The source of bacteriophages differs depending on their bacterial host. For example, phages against fish pathogens have been isolated from fish-farm water [38] and bacteriophages infecting human pathogens from human feces [39].

#### 2.1.4. Infection of Drug-Resistant Bacteria

Antibiotic-resistant bacteria cause serious diseases worldwide. The mechanism of action of bacteriophages is unlike that of antibiotics, making them effective against resistant pathogens. Bacteriophages infecting *P. aeruginosa*—a Gram-negative bacterium resistant to three classes of antibiotics—were isolated from sewage and wastewater and were effective against human infections without side effects [40]. In addition, bacteriophages have been used to control diarrhea caused by *E. coli* by oral administration of bacteriophages in pig models after challenging their host with the pathogenic bacteria [41,42].

#### 2.1.5. Increase in Number of Phage-Related Studies

Since the evolution of phages is established, phages can be developed as alternatives to antibiotics. Phages combined with antibiotics or other control agents have been used to control agricultural pathogens. In addition, bacteriophages can be applied as a liquid or cream. Brown et al. [43] showed that inclusion of a cocktail of phages in cetomacrogol inhibited *Cutibacterium acnes* (*Propionibacterium acnes*), the causative agent of acne. Bacteriophage studies led to characterization of enzymes that lyse bacterial cell membranes. Endolysins are expressed soon after bacteriophage entry and degrade peptidoglycan, resulting in cell death and release of new bacteriophages. Hence, research has applied endolysins extracellularly to kill pathogenic bacteria. Peng et al. [44] showed that recombinant phage endolysins AP50-31 and LysB4 are effective against *Bacillus anthracis* up to 3 days post-infection. However, endolysins are not active against Gram-negative bacteria because of the outer membrane, which prevents access to peptidoglycan. The recombinant endolysin LYSAB2 showed antibacterial activity against *Acinetobacter baumannii*. Endolysin LYSAB2 has a C-terminus containing an amphipathic α-helix that interacts with the outer membrane and an N-terminus that interacts with peptidoglycan, resulting in antibacterial activity. Therefore, endolysins are alternative antimicrobial agents.

### 2.2. Disadvantages of Bacteriophages

Phage infection begins with attachment to, and lysis of, the bacterial host. Therefore, for clinical use, bacteriophages must be virulent. Below we outline the disadvantages of phages as antibacterial agents.

#### 2.2.1. Bacteriophage-Resistant Bacteria

Evolution of bacteria promotes their survival in the presence of antibacterial agents, including bacteriophages. Bacteria resist lysis by bacteriophages by three strategies: prevention of phage absorption and phage DNA entry and abortion of the infection system [45].

#### 2.2.2. Blocking of Phage before Attachment

Infection begins with attachment of the phage to the bacterial cell. Hence, bacteria cells alter their surface receptors to inhibit attachment. For example, *S. aureus* produces protein A, which inhibits phage attachment [46]. Moreover, phase variation of bacteria results in production of surface adhesins, which alters their surface structure. *Bordetella* species use this strategy to prevent infection by phage BPP-1 [47].

#### 2.2.3. Production of Extracellular Matrix

Extracellular polymeric substances protect bacterial cells from harsh environments as well as from phages by preventing or delaying their attachment. However, some bacteriophages have evolved to recognize, and in some cases degrade, EPS. Hydrolases and lyases can be attached to the bacteriophage or released from lysed bacterial cells. For instance, bacteriophage F116 produces alginate lyase, enabling it to infect biofilm-embedded *Pseudomonas* spp. [48,49].

#### 2.2.4. Production of Inhibitors

Some bacteria produce protein inhibitors of bacteriophages, which block the phage entry receptor. We have pointed out different mechanisms below.

#### 2.2.5. Preventing Phage DNA Entry

Bacteria can resist infection by bacteriophages using superinfection exclusion (SIE) systems. These systems are usually encoded by phages that protect a lysogenized host from other bacteriophages. Membrane-anchored or -associated proteins block injection of phage DNA. Coliphage T4, which infects *E. coli*, has two SIE systems that inhibit DNA injection [50]. In Gram-negative bacteria, this system is encoded by the genes imm (immunity) and sp (spackle). Imm is localized to the membrane and blocks DNA injection into the cytoplasm by inducing conformational changes at the injection site. Sp inhibits bacteriophage DNA translocation and lysozyme. Phage lysozyme makes holes in the host cell wall, followed by phage DNA entry [51,52]. In Gram-positive bacteria, such as *Lactococus lactics*, the Sie 2009 system is localized to the bacterial cell membrane and inhibits entry of phage DNA [53].

#### 2.2.6. Restriction Modification Systems

Bacteria and other prokaryotic organisms have evolved a mechanism to defend against foreign DNA. Restriction-modification (R-M) systems are grouped into types I, II, and III, and are present in 90% of bacterial species [54]. Such systems involve a restriction endonuclease and methyl transferase. The latter methylates bacterial DNA at adenine and cytosine (specific sequences), conferring protection. The restriction endonuclease recognizes and cleaves foreign DNA [55]. However, bacteriophages have evolved anti-restriction strategies. Phages can remove recognition sites and/or reduce the number of recognition sites, preventing DNA cleavage. For instance, phage T7 has managed to cause distance on EcoRII sites within the phage genome, disabling the R-M system [56]. In addition, *Bacillus subtilis* and T-even phage genomes have been shown to have unusual base hydroxymethylcytosine, throwing off the restriction endonuclease [57]. Other phages employ proteins that bind restriction endonucleases; e.g., the overcome classical restriction protein of bacteriophage T7 blocks the nuclease [57,58].

#### 2.2.7. Bacterial Adaptive Immunity

Clusters of regularly interspaced short palindromic repeats (CRISPR) associated with Cas genes encode a bacterial adaptive immune system that confers resistance to foreign nucleic acids. CRISPR loci contain 21–48 base pairs of DNA repeats interspaced by non-repetitive spacers (26–72 bp). The upstream leader sequence contains a promoter encoding CRISPR RNA (crRNA) and recognition sequences for insertion of new spacers [59]. These areas occupy up to 1% of the bacterial genome, suggesting horizontal transfer. Phage resistance is mediated by addition of repeat-spacer units to susceptible bacteria. The effect of the CRISPR-CAS system occurs in three parts—adaptation, expression, and interference. In type I CRISPR/Cas, foreign DNA is recognized by Chi sites and the RecBCD machinery. The CAS1 and CAS2 protein complex extracts protospacers from foreign DNA and inserts new spacers between the CRISPR array and leader sequence, which later form crRNA with transcription of the CRISPR arrays. Next, crRNA and other required RNAs, such as trans-RNA, are expressed. In the type I interference step, Cas proteins are associated with crRNA at complementary sequences in the target DNA. However, a short sequence motif upstream of target DNA known as the protospacer adjacent motif (PAM) completes binding of the Cas complex. The PAM facilitates recognition of self from non-self-DNA by forming an R-loop structure between the dsDNA and crRNA. After recognition of the Cas complex and target site, Cas3 is activated and initiates DNA degradation [60,61]. Type II CRISPR/Cas systems encode a trans-encoded-cr RNA, which forms a complex with the Cas9 protein. This complex cleaves DNA near the PAM sequence. In type III CRISPR/Cas, Cas6 produces an R loop with pre-crRNA, which subsequently complexes with Cas10 and CSM (csm or cmr), resulting in a mature crRNA [62,63]. Type III does not need a PAM sequence; the csm/cmr complex attaches to the complementary target DNA. Type III systems require transcription of target DNA to single-stranded RNA complementary to crRN [64,65].

Bacteriophages have evolved to survive and infect bacterial hosts. Adaptation of phage fragments in a bacterial genome, the initial step for adaptive immunity, can be horizontally transferred to other bacteria. However, bacteriophages with different genotypes, a phenomenon known as mosaicism, maintain their infectivity in the host. In addition, genomic analysis of bacteriophages has indicated extensive recombination and blending of sequence motifs. Changes in sequence motifs not only provide a new infection pattern but also enable bacteriophages to evade the adaptive immunity mediated by CRIPSR [66]. For instance, Paez-Espino et al. [67] reported that the mutation rate in the bacteriophage genome is higher than in the host genome, resulting in persistence of phage. Hence, to overcome bacterial adaptive immunity bacteriophages adopt novel mechanisms and evolve to kill their bacterial host.

## 3. Approaches to the Application of Phages

### 3.1. Phage Therapy

The use of bacteriophages as therapeutic agents for controlling bacterial infections in animals and humans is called phage therapy. This practice started on a laboratory scale using single phage strains in animal models, which was called monophage therapy. Monophage therapy was mostly used to prove the concept of phage therapy during the development of phages in experimental models. Clinical trials of monophage therapy mainly consider bacteriophages with broad host ranges and phages with the ability to infect multidrug resistant pathogens. Jeon et al. [68] showed that the use of bacteriophages infecting carbapenem-resistant *A. baumannii* in a mouse model effectively reduced lung infections. A major hurdle to monophage therapy is the rapid development of phage-resistant bacteria. As the phage and host coevolve naturally, the application of single phages may be insufficient to control major diseases in the long term. Therefore, the use of a combination of phages (polyphage therapy) is common. Schmerer et al. [69] showed that the use of two phages together resulted in more bacterial lysis compared to single phages. In this strategy, multiple stains of phages that target one or multiple bacterial strains are used to control disease. A cocktail of multiple lytic bacteriophages not only improves phage therapy but as new bacteria develop resistance to one phage, other bacteriophages continue to control the disease. However, the preparation and purification of bacteriophages in cocktail form leads to longer, more complex procedures. To increase the efficiency of phage cocktails, phages with high bactericidal activity and bigger burst sizes are typically selected [70]. The final products are mostly a combination of few phages that are applied sequentially. The application of phages continuously instead of concurrently reduces the bacteria population and if new resistant bacteria appear the phages can be replaced with new infective phages. Long-term treatment with phages can also keep the bacterial population low, enabling action of the immune system. For instance, sequential use of phages against *P. aeruginosa* was more successful than simultaneous application [71].

The first phage therapy used in humans was serum therapy against pneumococci and diphtheria, wounds, and other external infections. In 2009, the first trial examined phage therapy against *S. aureus* and *P. aeruginosa* [72]. Wright et al. [73] demonstrated that the administration of phages in at least two phases improved chronic otitis in 24 patients. A 2-year study of a phage treatment called “Phagoburn”, which evaluated the effectiveness of a phage cocktail against *P*. *aeruginosa* on burns, showed a reduction of pathogens in the wounds [74]. These studies and studies of the oral [75] and intravenous application of phages indicate that phage therapy can be a reliable strategy. The overuse of antibiotics in animal farming and aquaculture has led to marked increases in resistant pathogens in meat for human consumption. These antibiotic-resistant bacteria from animals can affect humans [76]. It is important to note that phage therapy is not limited to humans but has also been proposed for fighting pathogenic bacteria in animal agriculture. Silva et al. [77] showed that bacteriophages can prevent *Vibrio anguillarum* infections in zebrafish larvae. Phage treatment of chickens infected with *Campylobacter* controlled bacterial infection within 5 days, although the time may depend on the phage and pathogen concentrations [78]. These and other studies of successful phage treatment support the use of phages on larger scales (Table 1).

### 3.2. Phage Biocontrol

The use of phages as a biocontrol against food and plant pathogens is called “phage biocontrol”. The food production industry has a long history of producing natural foods to increase the quality of food. As a biocontrol, phages can kill pathogenic bacteria without harming beneficial microorganisms. Their abundance and the widespread contact of phages with humans make them completely natural controls. Phages are present in fermented foods [99], vegetables, and other unprocessed foods and are less likely to raise an immune response in food consumers [100]. By contrast, while antibiotics enhance the quality of food, antibiotic-resistant bacteria can transfer the antibiotic-resistance genes to humans or other consumers and lead to disease [101]. Studies to develop phage biocontrol techniques have obtained noteworthy results. For example, bacteriophages infecting *L. monocytogenes* [101], *Salmonella* [102] and *E. coli* [103] have been developed and used safely on a commercial scale. Despite the increase in phage biocontrol, factors limiting the protective effects of phage biocontrol are important. Phage sensitivity to temperature, pH, and chemicals can be a hurdle to phage treatment. Guillier et al. [104] showed that environmental conditions affected the phage biocontrol of *L. monocytogenes*. The phage infection cycle can also interfere with phage protection and in some cases cause pathogenic hosts to become more virulent. The lysogenic phage CTXΦ, which infects *Vibrio cholerae*, carries genes that encode cholerae toxin [105]. The efficacy of phage treatment is also dependent on food processing, storage, and chemical or physical interventions. Generally, processed foods are treated with chemicals and preservatives that change the pH, altering phage titers. Food matrices play an important role in phage-host interactions. Solid foods are unlikely to facilitate virus-host interactions. The phage titer needs to be high enough to interact with low concentrations of the host in the same environment. However, the replication and growth of bacteria on food is highly dependent on the type of food, which is a major concern in phage-host interactions.

The control of plant pathogenic bacteria using phages has been studied since the discovery of phages. Phages have effectively controlled diseases caused by *Pseudomonas tolaasii* [106], *E. amylovora* [107], *R. solanacearum* [108] and *X. axonopodis* [109]. Phage products are produced commercially and used in integrated management strategies. However, like other phage applications, phage biocontrol of plant pathogenic bacteria faces challenges. Generally, phage-resistant bacteria must be considered in all stages of phage application. The large-scale phage control of pathogenic bacteria in farms or greenhouses can facilitate the rapid spread of resistant bacteria [110]. In addition, Environmental parameters such as pH, temperature, and ultraviolet (UV) light are more critical when phages are applied to plants. For example, sunlight, which includes UV-A and UV-B, is strongly negatively associated with phage survival. UV light significantly reduces titers of the phage ΦXacm and its ability to control bacterial spots in tomato plants [111]. In addition, high temperatures generally have deleterious effects. The phyllosphere temperature has a broad range and continuous long-term heat radiation from the sun increases the chance of killing bacteriophages. Desiccation, copper bactericides, and other environmental factors are also important in phage application and can reduce treatment efficiency. The proper timing of phage application to plants is required to maintain a sufficient phage population to control pathogens. Balogh et al. [112] demonstrated that phage application in the early evening is most effective for controlling bacterial spots in tomato. Strategies for improving phage application include using phages formulated with skim milk [94] and application at specific times of the day or during specific disease stages (Table 1).

### 3.3. Integrated Phage Application

The control of pathogenic bacteria is limited by bacterial resistance to control agents, such as antibiotics. The combination of bacteriophages with other control methods has been proposed for the effective control of bacterial diseases. While the resistance of bacteria to bacteriophages, antibiotics, and other control methods limits the control effect of each method, the combination of a harpin-phage with acibenzolar-S-methyl on tomato bacterial spot significantly reduced the severity of the disease [113].

### 3.4. Bacteriophages and Biofilms

The attachment of bacteria to surfaces and each other in a matrix called a biofilm enables bacterial survival. This matrix consists of proteins, polysaccharides, and water between the aggregations of cells. A biofilm not only helps bacteria hide from the immune system but also facilitates horizontal gene transfer between bacteria. Bacteria can also overcome the lack of nutrients in the environment by altering their gene expression within the biofilm. These adaptations help make bacteria resistant to antibacterial agents, which are inactivated in the biofilm [114].

Unlike antibiotics, which cannot cross the biofilm, phage interactions with bacteria help them to infect bacteria within the biofilm. Once bacteriophages recognize a surface bacterium in a biofilm they can continue to infect and kill it and produce thousands more bacteriophages. In the laboratory, phages carrying surface enzymes that degrade bacterial polysaccharides produced by both Gram-negative and Gram-positive bacteria produce a halo-type plaque, indicating degradation of LPS and bacteria small association to keep the biofilm within the spread bacterial population [115].

Phages can pass through bacterial biofilms in two major ways to reach their target and propagate. In the first way, bacteriophages are initially involved in killing a few host bacteria. Then, due to the high number of bacteria compared to phages in a biofilm, the bacteriophages spread and reduce the bacterial population, so-called “active penetration”. Alternatively, bacteriophages interact with biofilms using enzymes to hydrolyze extracellular polymeric substances, destroy the bacterial cell capsule, and interact with and lyse the bacterial cell wall [116,117,118].

### 3.5. Role of Bacteriophages in Bacterial Communities

Bacteriophages play an important role in the evolution of the microbial community. Phages are high in number with a very smaller size compared to bacteria. Phage and prey interaction initiates an evolutionary process where the diversity of microbial communities are mainly affected [119]. Changes in the individual bacterial species have direct effects on larger bacterial communities [120]. Wang et al. [88] showed that bacteriophage treatments against *R. solanacerum* not only decrease the incidence of the bacterial wilt disease in the tomato plant, but also a higher diversity in the bacterial community of rhizosphere can be seen. The potential mechanism is that the reduction of one bacterial population reduces the competitiveness for other bacterial populations resulting in a higher diversity in the environment. In addition, a slow growth rate of phage-sensitive bacteria slightly increases the phage resistance population in the same environment. However, to stay resistant to the bacteriophages, bacteria undergo a costly change that lowers their fitness to the environment. The resistant bacteria often trade the genes such as phage receptors genes with an important role in bacteria nutrient acquisition, mortality, and even virulence [121]. On the other hand, bacteriophages are often narrow host range that similar to other viruses, in order to keep their re-generation, they do not completely kill the host cells. This may explain the lysogenic state of bacteriophages where their life cycle continues within the genome of the host. Lysogenic bacteriophages or temperate bacteriophages introduce novel genes to the bacterial host. They start the superinfection immunity in the bacteria by eliminating the sensitive strains in the environment. The temperate phage also plays a major role in horizontal gene transfer (HGT) in the bacterial host [122]. Like mobile genetic elements, the acquisition of prophages, improves the contemporary evolution of bacteria. This evolution can either enhance the growth of bacteria or increase their chance to interact with other microbial communities in the environment.

Bacterial interaction mediated with bacteriophages increases the chance of the community to adapt to their environment. Bacteriophages can be considered as agents that keep the balance in the bacterial community. They can cause an increase or decrease in the population of certain bacterial species where maximum adoption of the bacterial community gives a direction to this balance. However, bacterial communities are only one part of the multiple microbial community in nature where other microorganisms such as fungi play a major role.

### 3.6. Future Aspects of Bacteriophage Application

The emergence of antimicrobial-resistant bacteria has promoted biocontrol strategies to combat bacterial diseases. Bacteriophages interact with the bacterial host and are involved in the evolutionary pathways of the host. Therefore, they provide great potential for synergistic application of phages and antimicrobial agents [69,123,124]. However, our knowledge about phages invoking evolutionary pathways in the host is limited and continuous research and studies are essential. In addition, the abundance of bacteriophages indicates their important role in the evolution of bacteria [1,17,125]. In fact, the bacterial host has developed resistant mechanisms against bacteriophage infection while effective counter-strategies by bacteriophages avoid the antiviral strategies. These arms races between phage and bacteria gave us a clear insight that phages are a reliable control method but not a perfect one. In addition, this information emphasizes that phages are active members of nature. The current understanding of antimicrobial-resistant bacteria raises concerns about control strategies. Overall it is predicted that the application of phages will not be the only possible strategy. Combined control strategies such as phage-antibiotics therapies will be developed and used in the future.

## 4. Conclusions

Bacteriophages, small in size with an abundant number on Earth have a great biological impact. In the past century scientists have shown that phages are important in the natural environment [126]. The use of phage as an alternative to antibiotics is an important tool and novel approaches towards understanding of phages are highly necessary. Therefore, in this review we introduced and reviewed the current ideas about nature of bacteriophages and successful use of bacteriophages against pathogenic bacteria. Information of this study can provide a direction for further research work on bacteriophages and pave the way for further application of phages.

## Figures and Tables

**Table 1:** Overview of application of bacteriophages in clinical and non-clinical studies.

Bacterial Pathogens	Host	Application Method	Control Effect of Bacteriophage	References
*Klebsiella pneumoniae*	Human, Skin	Phages were administered intraperitoneal 10 min after bacterial challenge mouse model	vB_KpnS_Kp13, effective against all Verona integron-encoded metallo-β-lactamase (VIM) producing *K. pneumoniae* isolates originating from hospital samples.	[79]
*Aeromonas hydrophila*	Fish, poikilothermy animals	Phage applied to *A. hydrophila-* challenged mice which hearts, livers, spleens, lungs and kidneys were collected for determination of bacterial loads	The phages G65 and Y81 showed considerable bacterial killing effect and potential in preventing formation of *A. hydrophila* biofilm; and the phages G65, W3 and N21 were able to scavenge mature biofilm effectively.	[80]
*Pseudomonas aeruginosa*	Human, lungs	Bacteriophage combination given via intravenous (IV) and inhaled routes to 67-year old male bilateral lung transplant recipient (LTR) who developed recurrent episodes of multi-drug resistant (MDR) *P. aeruginosa*	Complete control of disease By Bacteriophage combinations (BC).	[81]
*Listeria monocytogenes*	Human, Food pathogen	Phage treatment within a minute of contamination with a *L. monocytogenes* on ham.	Phage treatment kept *L. monocytogenes* below or at the detection level of 1 CFU/g after 28 (low treatment level) and after 42 days (high treatment level) whereas in the control levels exceeded 1 × 10^2^ CFU/g already after 14 days.	[82]
*Salmonella* Spp.	Human, Food pathogen	Combined application of bacteriophages and ultraviolet light applied on ground beef	Bacteriophages (S16 and FO1a) and ultraviolet light (UV) individually decreased approximately 1 log CFU/g. Combination of both showed to decrease twice the individual application.	[83]
*Vibrio parahaemolyticus*	Shrimp	Phage therapy (single phages and cocktails) on hatching and survival of brine shrimp (*Artemia franciscana*) cysts and nauplii exposed to pathogenic strains of *V. parahaemolyticus* and *V. harveyi*	100% hatching in Shrimp Cyst in 13 host compare to 40% hatching cyst in non-treated groups.	[84]
*Staphylococcus aureus*	Humans, organ infection	13 patients with severe *S. aureus* infections were intravenously administered three *Myoviridae* bacteriophages (AB-SA01)	Intravenously injected bacteriophages AB_SA01 control *S. aureus* infection in 13 patiens.	[85]
*Campylobacter jejuni*	Chicken	Various MoI was applied on 3 groups of broilers and *C. jejuni* was enumerated in cecal contents after 40 days.	Reductions in Campylobacter counts were statistically significant in phage treatments with MOI 0.1 compared to the control group.	[86]
*Propionibacterium acnes*	Human, skin (wounds)	Phage formulated in cetomacrogol cream aqueous for application. Mice were injected with phages after injection of *C. acnes.*	Phage treatments applied to mice with multi-drug-resistant (MDR) *C. acnes*-induced skin inflammation resulted in a significant decrease in inflammatory lesions.	[87]
*Ralstonia solanacearum*	Tomato plant	Phage treatment applied on the soil of tomato plant	Increasing the number of phages in combinations decreased the incidence of disease by up to 80% in greenhouse and field experiments during a single crop season.	[88]
*Pseudomonas syringae*	Cherry plant	Bean plants and cherry plants sprayed with the pathogenic bacteria and after a day they were sprayed with the selected bacteriophages	Phages could effectively reduce disease progression in vivo, both individually and in cocktails, reinforcing their potential as biocontrol agents in agriculture.	[89]
*Pectobacterium atrosepticum*	Potato plants	Tuber maceration with the pathogenic bacteria followed with the phage treatments	Use of the phage cocktail reduced both disease incidence and disease severity by 61% and 64%, respectively, strongly indicating that phage biocontrol has the potential to reduce the economic impact of soft rot in potato production.	[90]
*Xanthomonas euvesicatoria*	Pepper plant	Pepper crops were inoculated with the pathogenic bacteria and phages were sprayed on four leaf stage plants	Foliar applications of the unformulated KΦ1 phage suspension effectively controlled pepper bacterial spot compared to the standard treatment and the untreated control.	[91]
*Xanthomonas campestris pv. Campestris*	Brassicaceae (Cruciferae) plant	Bacteriophage was sprayed on the bacterial challenged plants	Effect of the Xccφ1 phage treatments on Xcc disease severity showed complete reduction in disease symptom V-shaped chlorotic to necrotic foliar lesions.	[92]
*Xyella fastidiosa*	Grapevines	Grapevines injected with bacteriophages	Grape plant treated with bacteriophage cocktail showed no development of Pierce’s Disease symptoms after 4 weeks compare to the control group which showed leaf scorching symptoms.	[93]
*Xanthamonas axonopodis PC. Citri*	Orange	Weekly spray if phages in citrus nursery	Treatment of phages in Valencia oranges showed disease progress inhibition in 3 various trial.	[94]
*Erwinia amylovora*	pear apple trees	Phage application on applied blossom and pear fruit slice	Three phage isolates (ΦEaH2A, ΦEaH5K and ΦEaH7B) significantly reduced bacterial multiplication and fire blight symptoms as compared to untreated controls.	[95]
*Pseudomonas tolaassi*	Mushrooms	Phages were applied on the mushroom tissue using pitting test	Phages can sterilize pathogenic bacteria in mushroom tissues as well as be useful for the biological control of brown blotch disease.	[96]
*Xanthomonas axonopodis pv.alli*	onion	Bacteriophages were sprayed on the plant leaves	Phage Φ31 reduced disease symptoms provided a significant increase in crop yield. Phage showed similar control effects compared to bactericides.	[97]
*Acidovorax citrulli*	Watermelon, Cucurbitacea	A seed coating method was used to control bacterial disease	Bacteriophage ACP17 and ACPWH were able to protect watermelon seeds and inhibit BFB symptoms.	[98]

## Data Availability

Not applicable.
